# Whole-Genome Sequence of Brucella melitensis CIIMS-BH-2, a Biovar 2 Strain Isolated from Human Blood

**DOI:** 10.1128/genomeA.00079-18

**Published:** 2018-03-08

**Authors:** Pooja M. Kishnani, Ankita A. Tiwari, Smita S. Mangalgi, Sukhadeo B. Barbuddhe, H. F. Daginawala, Lokendra R. Singh, Rajpal S. Kashyap

**Affiliations:** a Research Laboratory, G. M. Taori, Central India Institute of Medical Sciences (CIIMS), Bajaj Nagar, Nagpur, India; b Department of Microbiology, BLDEU's Shri B. M. Patil Medical College, Bijapur, Karnataka, India; c ICAR–National Research Centre on Meat, Hyderabad, India

## Abstract

Brucella species are the etiological agent of brucellosis in humans and animals. Here, we report the whole-genome sequence of Brucella melitensis strain CIIMS-BH-2, belonging to biovar 2. The draft assembly of CIIMS-BH-2 is 3.31 Mb in size, with 57.2% G+C content.

## GENOME ANNOUNCEMENT

Brucellosis is a zoonotic disease caused by Brucella species. Brucella spp. are Gram-negative, nonmotile, and facultative intracellular coccobacilli. Brucellosis in humans is characterized by undulant fever, arthritis, and gastrointestinal complications. It also causes a negative impact on livestock and public health (1). The classical Brucella species are divided into various groups depending on pathogenicity, phenotypic characteristics, and host preference. As of today, the genus Brucella consists of the following 12 species: B. melitensis (primary host, sheep and goats), B. abortus (cattle), B. suis (swine), B. canis (dogs), B. ovis (sheep), B. neotomae (wood rats), B. ceti (dolphins, porpoises, and whales), B. pinnipedialis (seals), B. microti (voles and foxes), B. inopinata (humans), B. papionism (baboons), and B. vulpis (foxes) (2, 3).

Though Brucella spp. lack classical virulence factors, they have the ability to adapt to various environmental conditions (4, 5). The mechanism driving intracellular invasion and persistence is still unknown or poorly understood. Thus, a complete genome analysis of Brucella species is the primary step for gaining knowledge on different mechanisms. For better understanding of Brucella virulence factors, comparative genomic analysis will enable the development of novel therapeutics and preventive strategies for brucellosis in humans and animals. A high seroprevalence of human brucellosis has been reported from various parts of India. Presently, very little information from the genome is available (6).

In the present study, whole-genome sequencing was carried out for the Brucella melitensis strain designated CIIMS-BH-2, an isolate from human blood. For whole-genome sequencing, the genomic DNA was isolated by using the Qiagen genomic DNA extraction kit from freshly grown culture. Libraries were constructed by using a paired-end library (2 × 100 bp) using version 2 chemistry. The sequencing of the isolate was performed on an Illumina HiSeq 2500 platform. The *de novo* assembly was carried out using SPAdes (version 3.10.1) (7). The sequencing resulted in 18,678,322 reads (583-fold read coverage). The draft assembly resulted in a genome size of 3,311,210 bp, with a G+C content of 57.2%. The average contig coverage was 295-fold. The genome sequence was annotated by using the Rapid Annotations using Subsystems Technology (RAST) server and the NCBI Prokaryotic Genome Annotation Pipeline (released 2013).

The 16S rRNA gene sequence of CIIMS-BH-2 revealed 100% similarity to that of Brucella melitensis 16M. As predicted by RAST, the CIIMS-BH-2 genome consists of 3,216 open reading frames (ORFs), including 3,113 protein-coding sequences with 67 RNAs (8). Further, genomic analysis reveals genes involved in pathways responsible for virulence and defense mechanisms. This includes genes responsible for antibiotic and toxic compound resistance, invasion, and intracellular survival. The comparative genomic analysis would be useful for the development of new diagnostic tools for brucellosis.

### Accession number(s).

The Brucella melitensis CIIMS-BH-2 genome sequence was deposited in the GenBank database under accession numbers CP025680 and CP025681.
